# The Effects of Acute Temperature Changes on Transcriptomic Responses in the Liver of Leopard Coral Groupers (*Plectropomus leopardus*)

**DOI:** 10.3390/antiox14020223

**Published:** 2025-02-15

**Authors:** Yilan Guo, Chaofan Jin, Cun Wei, Kangning Zhong, Yurui Gao, Peiyu Li, Zhe Qu, Zhenmin Bao, Bo Wang, Jingjie Hu

**Affiliations:** 1 MOE Key Laboratory of Marine Genetics and Breeding, College of Marine Life Sciences, Ocean University of China, Qingdao 266003, China; guoyilan@stu.ouc.edu.cn (Y.G.);; 2 Key Laboratory of Tropical Aquatic Germplasm of Hainan Province, Sanya Oceanographic Institution, Ocean University of China, Sanya 572025, China; 3 Southern Marine Science and Engineer Guangdong Laboratory, Guangzhou 511458, China

**Keywords:** temperature, *Plectropomus leopardus*, liver, transcriptome, antioxidants, lipid metabolism

## Abstract

The leopard coral grouper (*Plectropomus leopardus*) is a commercially significant tropical marine species. With the ongoing effects of global climate change, increasing attention has been focused on leopard coral grouper’s susceptibility to extreme cold weather. This study investigates the effects of acute cold exposure and temperature recovery on the liver of *P. leopardus*. Histological observations and enzyme activity assays revealed that temperature fluctuations caused significant disruptions to normal liver physiology, including lipid accumulation and alterations in antioxidant levels. Transcriptomic analysis of liver tissue identified 2744 differentially expressed genes (DEGs) across three experimental groups: 25 °C (control), 13 °C (cold exposure), and rewarming at 25 °C (R-25 °C). Functional enrichment analysis revealed that these DEGs were significantly associated with biological processes such as lipid metabolism and antioxidant defense, as well as pathways related to metabolism, fatty acid biosynthesis, and ferroptosis. Furthermore, dynamic regulation of lipid metabolism, immune responses, and oxidative stress pathways was observed in response to both cold stress and rewarming. Notably, several redox-related DEGs were identified, and their interactions with lipid metabolism were further explored. Additionally, representative DEGs associated with antioxidants and lipid metabolism, such as *got1*, *gpx1a*, *gpt*, and *g6pcla.2*, were validated by qRT-PCR and fluorescence in situ hybridization (FISH). Taken together, this study provides a systematic analysis of the effects of acute cold exposure and temperature recovery stress on the liver of the leopard coral grouper, laying the groundwork for further research on the temperature stress responses in teleost species.

## 1. Introduction

Water temperature is one of the most critical environmental factors influencing the growth, reproduction, and survival of aquatic organisms [1]. As global climate change intensifies, the impact of temperature fluctuations on aquaculture is becoming increasingly pronounced. Water temperature has a profound effect on the physiology, metabolism, and behavior of fish, and extreme temperatures—whether excessively high or low—pose significant threats to the health and productivity of aquaculture species. Fish are ectothermic organisms, and their body temperature is closely linked to the surrounding environment, meaning that their ability to adapt to changes in water temperature directly determines their growth and physiological functions [2,3,4,5]. When fish are exposed to acute unsuitable temperature conditions, such as extreme heat or cold, their health can be severely impacted, potentially leading to death [6,7,8]. For example, in Silver Barb (*Barbonymus gonionotus*), low temperatures can cause gill vascular lesions, hemorrhagic telangiectasia, and tissue swelling [9]. The lamellae of crucian carp (*Carassius carassius*) gills are embedded in a cell mass at low temperatures [10]. The intestine of the low temperature 9 °C group appeared to have broken mucosa; the lamina propria became vacuolated, resulting in the increase in intestinal permeability in the large yellow croaker (*Larimichthys crocea*) [11].

The liver, as a key metabolic organ in fish, performs essential functions such as synthesis, catabolism, and detoxification and plays a central role in regulating energy metabolism, immune responses, lipid synthesis, and antioxidant defense [12,13]. Temperature fluctuations significantly impact the physiological functions of the liver, with the organ playing a crucial role in responding to thermal stress [14,15]. Under both cold and heat stress conditions, the liver of fish adjusts its metabolic activities to adapt to environmental changes, thereby maintaining homeostasis [16,17]. In-depth research into the physiological response mechanisms of the liver to temperature fluctuations is crucial for understanding how fish maintain physiological stability under extreme temperature conditions, and it is an important direction in the study of fish temperature stress. In recent decades, numerous studies have investigated the effects of cold stress on fish livers from the insights of physiology and transcriptomic profiling. For instance, in Black Porgy (*Acanthopagrus schlegelii*), exposure to low temperatures of 5/10 °C leads to enhanced hepatic lipid synthesis, activation of gluconeogenesis pathways, and upregulation of DNA damage repair mechanisms [18]. In gynogenetic mrigal carp (*Cirrhinus mrigala*), oxidative phosphorylation, proteasome activity, and the citric acid cycle are upregulated in the liver, while genes associated with immune pathways are downregulated [19]. In tilapia (GIFT strain, *Oreochromis niloticus*), cold stress triggers hepatic gluconeogenesis and inhibits lipolysis, providing energy to the body while reducing protein synthesis efficiency [20].

The leopard coral grouper (*Plectropomus leopardus*), a species belonging to the family Serranidae, subfamily Epinephelinae, and genus *Plectropomus*, is a tropical reef fish primarily distributed across the Indo-Pacific region [21]. Known for its vibrant coloration and nutritional value, it has become a beloved species in Southeast Asia and an economically important fish [22]. The suitable water temperature range for *P. leopardus* is between 22 and 30 °C, and any abnormal drop in sea or rearing water temperatures poses significant challenges to its survival and healthy growth [23]. To date, various studies have been carried out to investigate *P. leoaprdus* growth, disease resistance, and body coloration and provided valuable insights [22,24,25,26]. However, the systematic studies underlying the responses of *P. leopardus* to temperature fluctuations remain lacking. In this study, histological and enzyme activity assays revealed that temperature fluctuations significantly disrupted liver function, including lipid accumulation and changes in antioxidant levels. Transcriptomic analysis demonstrated the dynamic regulation of lipid metabolism, immune responses, and oxidative stress pathways was observed during both cold stress and rewarming. Notably, several redox-related DEGs were identified, and their interactions with lipid metabolism were further explored. Key DEGs associated with antioxidants and lipid metabolism, including *got1*, *gpx1a*, *gpt*, and *g6pcla.2*, were validated by qRT-PCR and FISH. This study provides a comprehensive analysis of the liver’s response to acute cold exposure and temperature recovery and provides valuable insights for the breeding of temperature-resilient *P. leopardus* varieties.

## 2. Materials and Methods

### 2.1. Ethics Statement

This study was carried out with the permission of the College of Marine Life Sciences, the Ocean University of China Institutional Animal Care and Use Committee on 10 October 2018 (Project Identification Code: 20181010).

### 2.2. Experimental Design and Sampling

**Figure 1:**
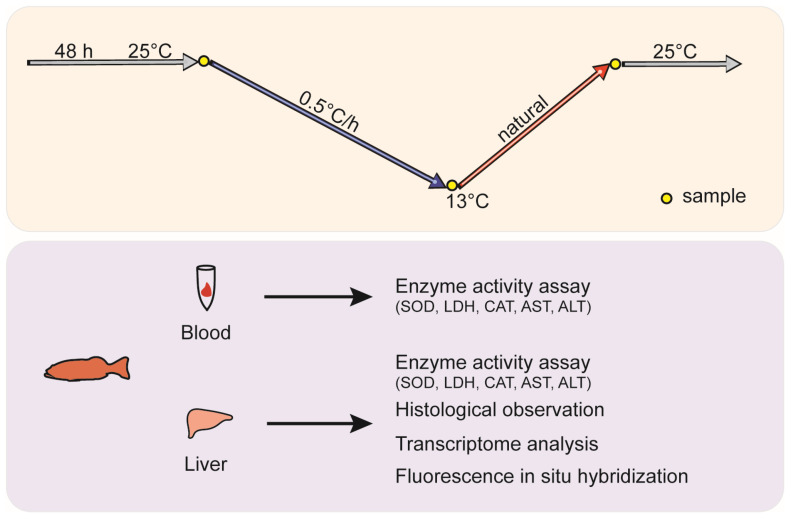
Experimental design.

Six-month-old leopard coral groupers with an average weight of 19.0 ± 2.1 g were provided by Chenghai Aquaculture Co., Ltd. in Dongfang City, Hainan Province, China. The 150 *P. leopardus* individuals were equally divided into three 100 L tanks. Using a chiller and a circulation pump, the water temperature was gradually reduced from 25 °C to 13 °C at a rate of 0.5 °C per hour over a 24 h period. The cooling process was then halted, allowing the water temperature to naturally return to 25 °C. The collected samples were categorized into three groups based on three specific sampling time points: the normal temperature group (25 °C), the low-temperature group (13 °C), and the recovery temperature group (R-25 °C). Sampling was conducted at 11 PM on the first day, 11 PM on the second day, and 11 AM on the third day. These sampling points are clearly indicated in Figure 1, providing a visual representation of the experimental timeline. Six individuals from each group were anesthetized with MS222. Blood samples were collected using sterile syringes and stored at 4 °C for enzyme activity assay. Part of the liver tissues were fixed by 4% paraformaldehyde solution and sequentially dehydrated by immersing them in 30%, 50%, 70%, 80%, and 90% ethanol, followed by absolute ethanol, for 2 h each and stored in 100% ethanol. The other part of liver tissue was treated with liquid nitrogen and stored at −80 °C for RNA extraction and enzyme activity assay (Figure 1).

### 2.3. Enzyme Activity Assay

After being incubated at 4 °C overnight, the blood samples were centrifuged at 3500 rpm for 10 min, and the supernatant was collected for further analysis. Liver tissues were weighed and homogenized in ice-cold 0.65% saline solution using a handheld homogenizer in an ice-water bath. The homogenate was centrifuged at 2500–3000 rpm for 10 min, and the supernatant was collected for subsequent assays.

Enzymatic activities of superoxide dismutase (SOD, WST-1 method, A001-3-2), lactate dehydrogenase (LDH, A020-2-2), catalase (CAT, A007-1-1), aspartate aminotransferase (AST, C010-2-1), and alanine aminotransferase (ALT, C009-2-1) in both serum and liver homogenate supernatants were measured using commercial assay kits from Nanjing Jiancheng Bioengineering Institute, Nanjing, China. In brief, supernatants were diluted to the appropriate concentrations and assayed in 96-well plates. Samples, enzymes, and substrates were added sequentially and incubated at 37 °C for 1–30 min. Absorbance values at 405–562 nm were measured using a multi-function microplate reader (BioTek Synergy H1, Agilent, Santa Clara, CA, USA). The enzyme activities were calculated based on standard curves generated during the assays.

### 2.4. RNA Extraction, cDNA Library Construction, and Sequencing

Total RNA of livers of three individuals from every group was extracted using TRIzol reagent (Invitrogen, Carlsbad, CA, USA) following the standard procedure of our lab. After removing genomic DNA, the concentration and quality of the total RNA were measured using a Qubit 4 Fluorometer (Thermo Fisher Scientific, Waltham, MA, USA) and 1% agarose gel electrophoresis. The RNA with high quality was utilized for library construction using the Illumina TruSeq RNA Sample Prep Kit (Illumina, San Diego, CA, USA) based on manufacturers procedures. Finally, the cDNA library was sequenced on the Illumina Nova-seq 6000 platform with PE150 strategies.

### 2.5. Transcriptomic Data Processing and Analysis

The raw reads were checked by FastQC v0.11, and clean data were generated after the removal of adaptors and low-quality reads using Trimmomatic v0.39. The clean reads were then mapped to the reference genome of *Plectropomus leopardus* (GenBank number: GCF_008729295.1) using HISAT2 v2.2.1, producing alignment files in BAM format. These BAM files were annotated, and counts per sample were obtained using the featureCounts program. Transcripts per million (TPM) values were converted from count values and used for correlation and principal component analysis (PCA). Counts were utilized for differential gene expression analysis with the standard of |log2FoldChange| ≥ 2, and *q*-value ≤ 0.05, using the Bioconductor package DESeq2. Venn plots were constructed using the ggplots package, and the expression heatmap of differentially expressed genes (DEGs) was plotted using ggplot2 and pheatmap packages. Gene Ontology (GO) annotation and KEGG enrichment analysis of DEGs were conducted using OmicShare tools (www.omicshare.com/tools, accessed on 8 October 2024). Bubble plots were generated using the Bioinformatics online platform for data analysis and visualization (https://www.bioinformatics.com.cn, accessed on 10 October 2024).

### 2.6. cDNA Synthesis and qRT-PCR

The cDNA synthesis of the total RNA of the livers of six individuals from every group was performed using All-In-One 5X RT MasterMix (abm, Zhenjiang, China). Gene-specific primers for qRT-PCR were designed using the NCBI Primer-BLAST tool (Table S1). The b2m gene was used as a reference gene for standardizing gene expressions. The qRT-PCR analysis was performed on a LightCycler 480 (Roche, Forrentrasse, Switzerland). The reaction mixture consisted of 10 μL BlasTaq 2× qPCR MasterMix (abm, Vancouver, BC, Canada), 2 μL cDNA, 0.5 μL of each primer (10 μM), and 7 μL nuclease-free H_2_O. The thermal cycling conditions included an initial enzyme activation at 95 °C for 3 min, followed by 40 cycles of denaturation at 95 °C for 15 s and extension at 60 °C for 1 min. The relative expression of the target genes was calculated using the 2^−ΔΔCt^ method.

### 2.7. HE Staining and Fluorescence in Situ Hybridization (FISH)

After dehydration, liver tissues preserved in absolute ethanol were transferred to a 1:1 mixture of xylene and absolute ethanol for 10 min. The tissues were then hyalinized twice in xylene, each time for approximately 5 min. The tissues were immersed in a 1:1 mixture of melted paraffin and xylene for 30 min, followed by two additional immersions in pure paraffin, each lasting 1 h, to ensure thorough infiltration of the paraffin into the tissues. Finally, the tissues were embedded in paraffin and cut into 5 μm thickness. The slices were floated on water at 42 °C to stretch, then lifted and dried at 37 °C. Then, the staining was conducted using haematoxylin-eosin staining kit (Solarbio, Beijing, China) and the results were observed and photographed by an Olympus BX43 microscope (Tokyo, Japan).

Primers for probe template amplification in FISH were designed using the NCBI Primer-BLAST tool (Table S2). RNA probes were synthesized using a biotin or digoxigenin (DIG)-labeled RNA labeling kit according to standard protocol. Subsequently, the FISH was performed as described previously. Briefly, the tissue sections were digested for 15 min with 2 μg/mL proteinase K and fixed with 4% paraformaldehyde. RNA probes were denatured to single strands before use. Pre-hybridization was performed at 56 °C for 3 h, followed by hybridization with 1 ng/μL RNA probes at 56 °C for 17 h. Unhybridized probes were washed off with saline sodium citrate. The liver sections were then incubated with streptavidin-Cy3™ and anti-digoxigenin fluorescein (from Avidin-Streptavidin) in the dark at 37 °C for 1 h. The cell nuclei were stained with 5 μg/mL DAPI diluted in PBS. After applying a drop of antifade mounting medium (P0126, Beyotime Biotech Inc, Shanghai, China) onto the sample, cover it with a coverslip to complete the mounting process. Finally, imaging was performed using an Olympus FV3000 confocal microscope (Olympus, Tokyo, Japan) and FV31S SW software version 2.6.

### 2.8. Statistical Analysis

All data were analyzed using IBM SPSS Statistics version 27.0 (SPSS Inc., Chicago, IL, USA). Differences were assessed by one-way analysis of variance (ANOVA). Results are presented as mean ± standard error of the mean (SEM), with a significance level set at *p* < 0.05. When significant differences were detected, Duncan’s multiple range test was used to determine differences between experimental groups.

## 3. Results

### 3.1. Effects of Temperature Stress on Hepatic Histology and Enzyme Activity in Serum and Liver

**Figure 2:**
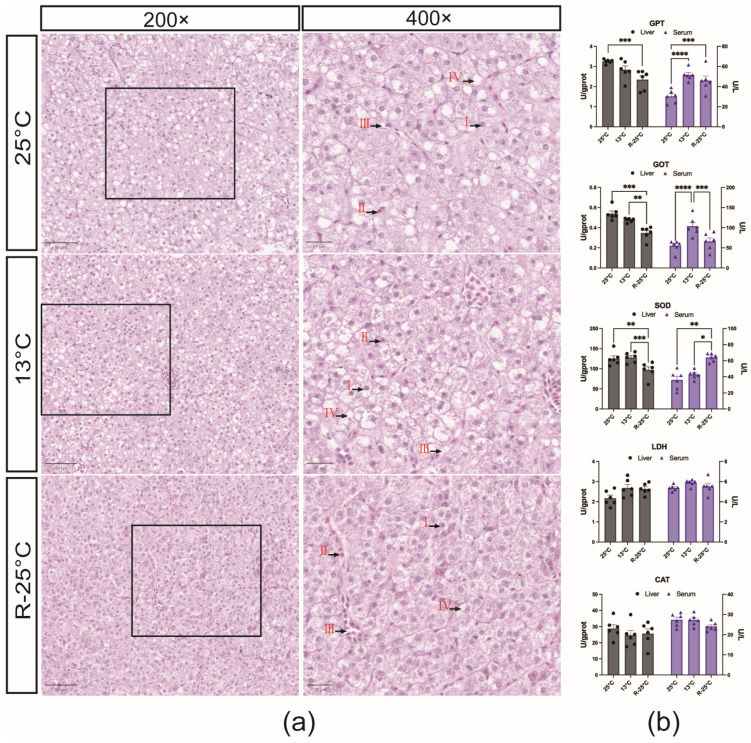
The hepatic histological alteration and dynamics of enzyme activities in response to acute temperature fluctuation. (**a**) Histological observations of liver from *P. leopardus* under low-temperature effects. The left image is at 200× magnification with a scale bar of 50 μm; the right image is at 400× magnification with a scale bar of 20 μm. I. Hepatocyte nuclei; II. Blood cells; III. Sinusoids; IV. Lipid droplets. No pathological changes were observed in the three groups. The number of lipid droplets within hepatocytes decreases sequentially in the 25 °C, 13 °C, and R-25 °C groups. (**b**) The enzyme activity alterations in the liver or serum in response to temperature stress in *P. leopardus*. Mean and SEM. The * (*p* < 0.05), ** (*p* < 0.01) *** (*p* < 0.001) and **** (*p* < 0.0001) indicated the significant difference between groups.

Histological analysis revealed that the liver cells were polygonal in shape and organized in a plate-like structure, with centrally located round nuclei. Liver sinusoids were evident, containing distributed blood cells. A slight reduction in fat accumulation within the hepatocytes was observed in both the low temperature (13 °C) and recovery temperature (R-25 °C) groups, compared to the normal temperature group (25 °C). No significant pathological alterations, such as sinusoidal congestion, hepatocyte degeneration, or tissue edema, were observed in any of the three groups (Figure 2a).

Serum enzyme activities of SOD, GOT, GPT, CAT, and LDH were measured in three parallel samples from the normal temperature group (25 °C), low-temperature group (13 °C), and recovery temperature group (R-25 °C). As shown in Figure 2b, no significant differences were observed in the activities of LDH and CAT among the three groups. However, significant differences were found in the activities of SOD, GOT, and GPT. These enzymes also showed corresponding activity changes in liver tissue homogenates. SOD, which plays a crucial role in cellular stress responses and metabolism, exhibited a significant upregulation in the R-25 °C group, with activities markedly higher than those in the normal and low-temperature groups. GOT and GPT activities are critical indicators of liver function. Our results demonstrated that both GOT and GPT were significantly elevated in the low-temperature group (*p* < 0.05), with GOT showing a marked decrease in the recovery group.

Additionally, the enzyme activities in the liver supernatants were also measured. As shown in Figure 2b. Significant changes were observed in the activities of SOD, GOT, and GPT among the groups, although the trends differed from those observed in serum. The activities of these three enzymes were generally downregulated in liver tissues, with the most pronounced decrease occurring in the recovery group.

### 3.2. DEGs Identification Among Three Groups Based on Transcriptomic Data

**Figure 3:**
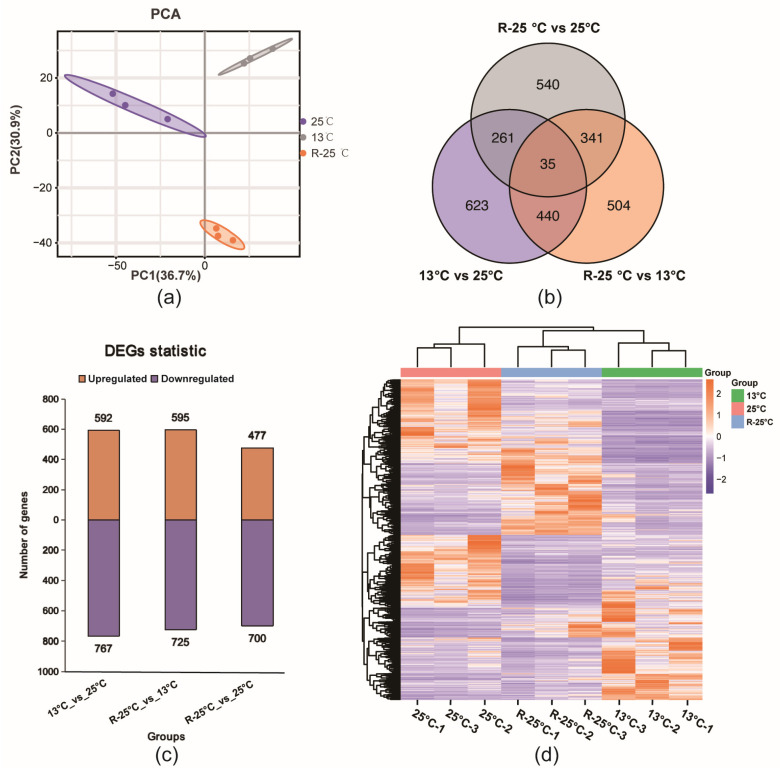
Comprehensive transcriptomic analysis of temperature effects on *P. Leopardus* in the liver. (**a**) PCA of all genes across nine individuals showing variance distribution. (**b**) Venn diagram of DEGs across three comparative groups. (**c**) Differential expression gene statistics: bar chart of the number of upregulated and downregulated genes between groups. (**d**) The expression heatmap of all DEGs in the liver.

Firstly, the PCA results revealed the samples from one group were clustered together, respectively (Figure 3a), indicating that the temperature fluctuation caused obvious changes in gene expression patterns. Subsequently, pairwise comparisons between groups were set up to perform DEGs analysis. As displayed in Figure 3b, a total of 2744 DEGs were identified from three comparison groups, among which, 35 DEGs were shared in all three comparison groups (Figure 3b) Subsequently, the number of upregulated or downregulated DEGs in each comparison group is concluded in Figure 3c. In addition, the expression heatmap of all DEGs also demonstrated that there was clear separation between groups in the gene expression profile (Figure 3d). More detailed information of DEGs was concluded in Table S3.

### 3.3. Functional Analysis of DEGs

**Figure 4:**
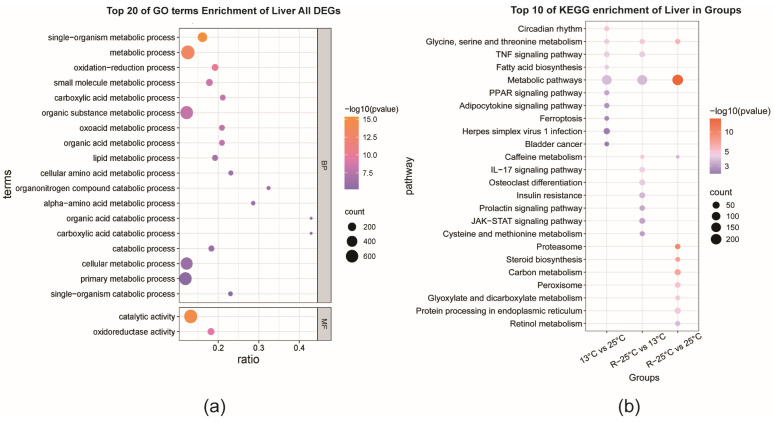
Functional enrichment analysis of all DEGs from the liver on *P. Leopardus.* (**a**) Top 20 GO terms. BP, biological process; MF, molecular function. (**b**) Composite bubble plot of top 10 KEGG enrichment pathways of DEGs from each comparison group.

To investigate the functional implications of genes exhibiting significant transcriptional changes in response to temperature variations in the liver of *P. leopardus*, GO and KEGG enrichment analyses were performed on all DEGs. The GO analysis revealed that among the top 20 significantly enriched terms, 18 were associated with biological processes (BP), and two were linked to molecular functions (MF). The majority of these terms were related to catabolic metabolism. Notably, both BP and MF categories demonstrated significant enrichment in oxidation-reduction processes, including “oxidation-reduction process” and “oxidoreductase activity”, as illustrated in Figure 4a.

DEGs from pairwise comparisons across three groups were then subjected to KEGG enrichment analysis. The results, depicted in Figure S1, highlighted that the most enriched categories across all comparative groups were related to metabolism and environmental information processing. Specifically, within the environmental information processing category, the signal transduction processes harbored the most enriched genes between the temperature differential groups (13 °C vs. 25 °C and R-25 °C vs. 13 °C). Conversely, in the temperature equivalent group (R-25 °C vs. 25 °C), the most enriched genes were associated with the metabolism category, particularly in the ‘global and overview maps’ processes. Then, the top ten significant pathways from each comparison group were concluded (Figure 4b). DEGs from all three comparison groups were related to ‘glycine, serine, and threonine metabolism’, as well as general ‘metabolic pathways’. Among these, the R-25 °C vs. 25 °C group showed the highest significance for metabolic pathways, indicating varying degrees of energy metabolism changes between the temperature shift groups. Compared to the 25 °C group, the 13 °C group showed enrichment in pathways related to ‘fatty acid synthesis’, ‘adipokine signaling’, ‘TNF signaling’, ‘PPAR signaling’, and ‘ferroptosis’. The R-25 °C group, compared to the 13 °C group, was enriched in pathways related to ‘insulin resistance’, ‘TNF signaling’, and ‘IL-17 signaling’.

**Figure 5:**
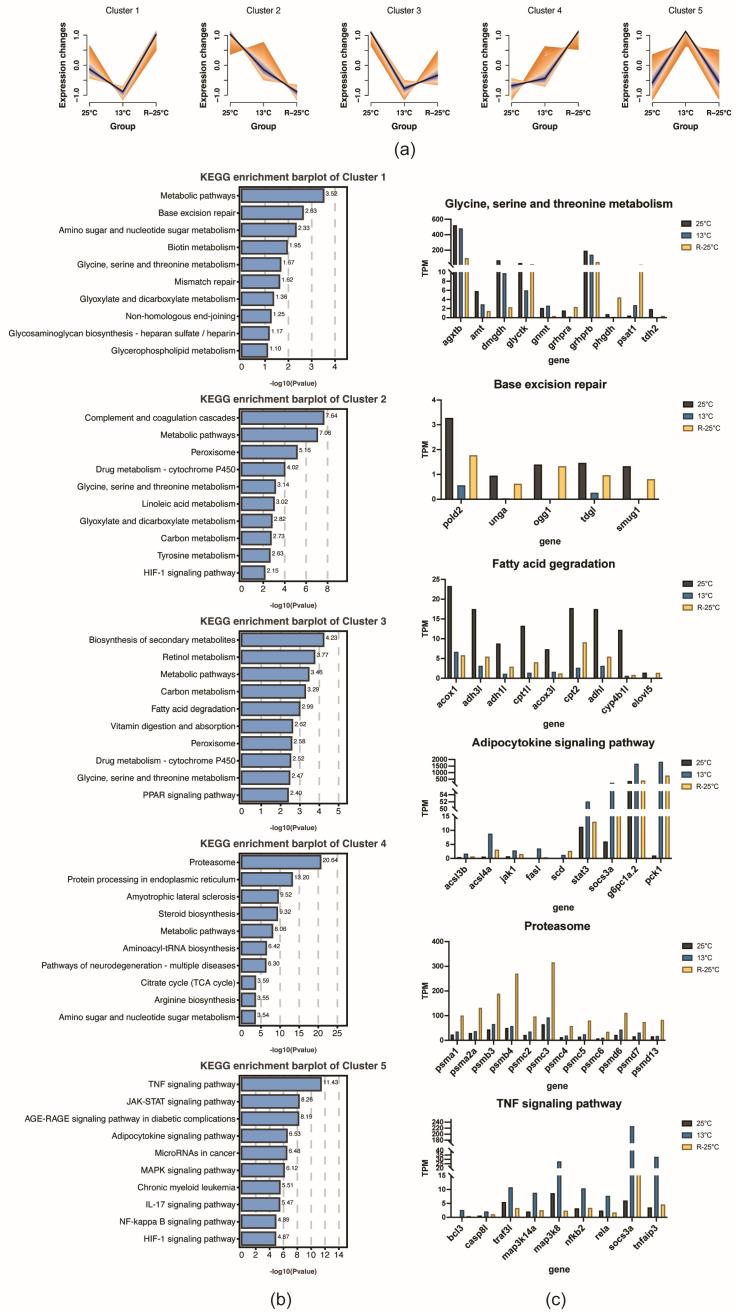
Trend analysis of DEGs and functional analysis in the liver of *P. Leopardus* under temperature changes. (**a**) Trend analysis of DEGs. (**b**) KEGG enrichment results of DEGs from five clusters. (**c**) Expression analysis of representative DEGs in key pathways under temperature changes.

To gain deeper insights into the dynamic regulation of the transcriptomic profile in response to temperature stress, the trend analysis for DEGs was performed, generating five distinct groups (Figure 5a). The results indicate that genes in Cluster 1 show decreased expression at 13 °C but were upregulated upon rewarming; GO enrichment results demonstrated that Cluster 1 genes were mainly associated with DNA repair and damage response (Figure 5b and Figure S2). DEGs in Cluster 2 exhibited a sustained decline during both cooling and rewarming, primarily associated with oxidative-reduction processes. In Cluster 3, gene expression was suppressed in response to temperature fluctuations and was strongly correlated with fatty acid oxidation. Genes in Cluster 4 were upregulated by temperature changes and were closely linked to proteasomal function and catalytic activity. In Cluster 5, genes were highly expressed during cooling and were predominantly involved in inflammation and immune responses. Furthermore, the expression dynamics of DEGs involved in representative pathways, including glycine, serine, and threonine metabolism, base excision repair, fatty acid degradation, adipocytokine signaling pathway, proteasome, and TNF signaling pathway, were concluded (Figure 5c). The results exhibited that DEGs related to fatty acid degradation, such as *acox1*, *adh1*, *cpt1l*, and *cyp4b1l*, were significantly downregulated after temperature changes. Proteasome-related genes showed significant upregulation upon rewarming. Additionally, TNF signaling pathway genes, such as *bcl3*, *rela*, *map3k8*, and *socs3a*, were significantly upregulated at 13 °C.

### 3.4. The Regulation of Redox Related DEGs Under the Temperature Fluctuations

**Figure 6:**
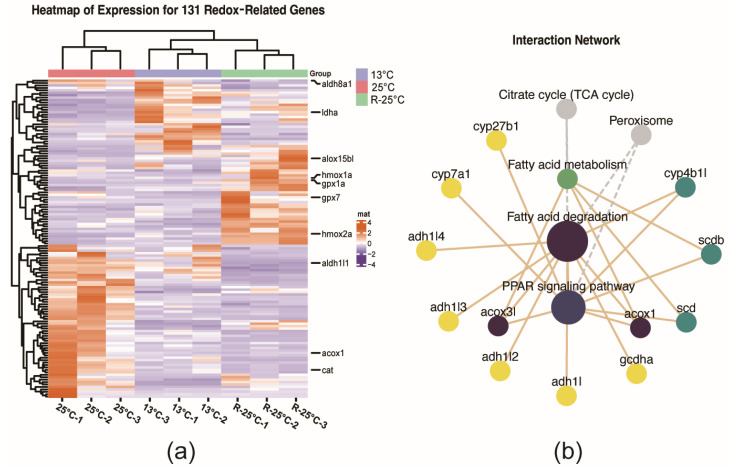
The regulation of redox DEGs under temperature fluctuations. (**a**) Heatmap of expression levels for 131 genes related to redox processes. (**b**) Network diagram between redox-related genes and fatty acid metabolism pathways.

In this study, a total of 131 DEGs related to redox were identified and the heatmap revealed their distinct expression profiles across different temperature conditions (Figure 6a). Notably, genes associated with liver enzyme activity, such as catalase (*cat*), glutathione peroxidase genes (*gpx1a* and *gpx7*), and lactate dehydrogenase (*ldha*), exhibit varying expression levels under these conditions. Subsequent KEGG pathway enrichment analysis demonstrated that these genes were implicated in various metabolic and synthetic pathways, including the ‘peroxisome’, ‘cytochrome P450 pathway’ and ‘PPAR signaling pathway’ (Figure S3). To investigate the relationships between redox-related genes and fatty acid metabolism pathways, we constructed a network diagram (Figure 6b). The analysis revealed that acyl-CoA oxidase 1 (*acox*1) and peroxisomal acyl-coenzyme A oxidase 3-like (*acox3l*) exhibited obvious connections with multiple fatty acid metabolism pathways.

### 3.5. Validation of Gene Expression Profiles

**Figure 7:**
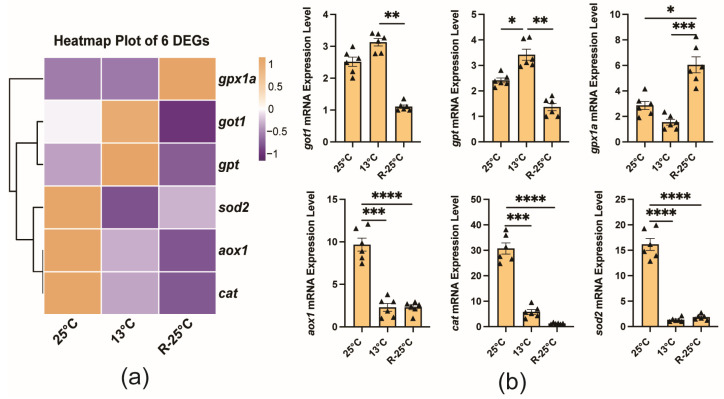
Validation of the expression patterns of representative DEGs by qRT-PCR. (**a**) Heatmap of transcriptomic expression (TPM) for six DEGs. (**b**) Quantitative PCR analysis for six DEGs. Mean and SEM. The * (*p* < 0.05), ** (*p* < 0.01), *** (*p* < 0.001), and **** (*p* < 0.0001) indicated the significant difference between groups.

Firstly, qRT-PCR analysis was performed to validate the expressions of several enzyme-related DEGs (*sod2*, *cat*, *gpt*, and *got1*), the glutathione peroxidase gene (*gpx1a*), and the aldehyde oxidase gene (*aox1*). The results indicated that the expression patterns of these genes in the liver were consistent between the TPM value and the qRT-PCR results (Figure 7). Specifically, *cat*, *aox1*, and *sod2* exhibited the highest expression levels in the control group, with a subsequent decline observed following temperature changes. In contrast, the expression levels of *gpt* and *got1* peaked at 13 °C, after which they decreased upon rewarming. The *gpx1a* gene showed the highest expression level in the R-25 °C group.

**Figure 8:**
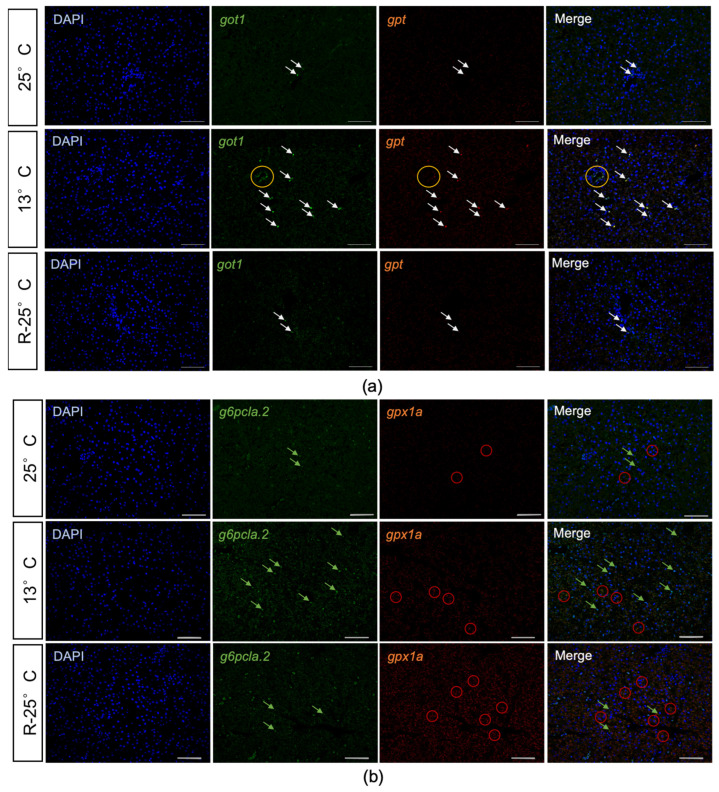
Validation of DEGs expression profiles by FISH. (**a**) FISH validation of the expressions of *got1* and *gpt* in *P. leopardus* liver across different temperature conditions. Arrows indicated the signals in the cell nucleus of *got1* and *gpt*. Circles indicated the concentrated signals in blood vessel walls of *got1* and *gpt*. (**b**) In situ expression of *g6pcla.2* and *gpx1a* in *P. leopardus* liver across different temperature conditions. Green arrows indicated the positive signals of *g6pcla.2*, while red circles highlighted the signals of *gpx1a*.

Furthermore, FISH was carried out to further validate the expression of DEGs. As shown in Figure 8a, the signals of *got1* and *gpt* were not obvious in the 25 °C group. In the 13 °C group, the expressions of *got1* and *gpt* in the hepatocyte nuclei were significantly increased. In the R-25 °C group, the expression levels of *got1* and *gpt* were reduced compared to the 13 °C group. Notably, in the 13 °C group, the nuclei of cells with high expression of *got1* and *gpt* exhibited irregular and non-circular shapes (indicated by arrows), and cells on the vascular walls showed concentrated signals of *got1* mRNA (indicated by circles). In addition, the DEGs related to glutathione peroxidase were also validated by FISH (Figure 8b).

In the 25 °C group, the positive signals of *g6pcla.2* were weak in the cytoplasm of hepatocytes. With a decrease in temperature to 13 °C, the signals became stronger and concentrated in the nuclei of hepatocytes. Upon rewarming, the expression of *g6pcla.2* decreased significantly. Conversely, the signals of *gpx1a* were intensified following temperature changes, reaching their highest level in the 25 °C group following rewarming.

## 4. Discussion

### 4.1. Low Temperature Leads to Expression Changes in Membrane Fluidity Maintenance-Related Genes

The relationship between membrane fluidity and temperature is generally inverse, with typically increasing membrane fluidity in response to decreasing temperatures and vice versa [27,28]. Existing research has shown that cell membranes maintain fluidity through mechanisms such as increasing the proportion of unsaturated fatty acids, altering fatty acid chain lengths, and regulating cholesterol content [29,30]. Our transcriptomic analysis revealed that exposure to 13 °C induced the enrichment of pathways associated with lipid metabolism in the liver of *P. leopardus*, including the PPAR signaling pathway, fatty acid synthesis, and adipocytokine signaling pathways. Under cold exposure, the expressions of genes related to fatty acid synthesis, such as acyl-CoA synthetase long-chain family member 4a (*acsl4a*), acyl-CoA synthetase long-chain family member 3b *(acsl3b*) [31], and fatty acid synthase-like (*fasnl*), were upregulated. This upregulation could promote the synthesis of long-chain fatty acids from acetyl-CoA. Additionally, the upregulation of *elovl5* might facilitate the elongation of fatty acid chains, providing appropriate substrates for subsequent desaturation processes [32]. Simultaneously, the expressions of genes related to fatty acid transport (such as *lpl* and *slc27a4*) were also upregulated, aiding in the transport of fatty acids to the sites of desaturation [33,34]. The *scd* gene encodes the SCD enzyme, which introduces the first double bond into stearic acid (C18:0), forming the monounsaturated fatty acid oleic acid (C18:1 Δ9c) [35]. Oleic acid, an important component of cell membranes, serves multiple critical functions within these membranes [36]. The upregulation of *scd* genes enhances the unsaturation of fatty acids, thereby maintaining membrane fluidity under cold conditions [37]. Conversely, the downregulation of genes involved in cholesterol metabolism (such as *cyp7a1* and *cyp27*) could reduce cholesterol metabolism, contributing to maintaining membrane stability at lower temperatures [38]. There were studies suggesting that cholesterol can maintain membrane stability at low temperatures [30]. Furthermore, several studies have indicated that under cold conditions, a decrease in fatty acid oxidation can make more fatty acids available for membrane synthesis, thereby maintaining membrane fluidity [39,40,41]. Our results also show a reduction in the expression of genes related to fatty acid oxidation (such as *cpt1*, *cpt2*, and *cyp4a1*), indicating a decrease in fatty acid oxidation and allowing more fatty acids to be used for membrane synthesis, thus maintaining membrane fluidity. While our transcriptomic data suggest potential shifts in metabolic pathways, further validation is necessary to fully understand these changes. Notably, this study did not measure the composition and ratios of fatty acids in the liver, introducing certain limitations to our findings.

### 4.2. Low Temperature Promotes Upregulation of Hepatic Gluconeogenesis Related Genes

Under low-temperature conditions, the hormonal levels and gene expressions in certain fish are significantly affected, leading to decreased appetite and reduced food intake. For example, under cold temperatures, the mRNA levels of the leptin gene (*lep-a* and *lep-b*) increased in the livers of goldfish [42]. In Atlantic cod, low temperatures inhibited food intake, an effect mediated by an increase in the expression of cocaine- and amphetamine-regulated transcript (CART), an anorexigenic factor [43]. Ayelén Melisa Blanco [44] has also discussed the role of leptin in regulating feeding behavior in teleost fish, proposing that when leptin binds to its receptor lepR, it activates the Jak/STAT intracellular signaling pathway. This activation leads to downregulation of orexigenic gene expression and upregulation of anorexigenic gene expression in the nucleus, ultimately resulting in decreased food intake [44]. Our transcriptomic analysis revealed a significant increase in the mRNA levels of leptin in the liver following temperature changes, which might result in reduced food uptake and energy lacking.

*g6pc1a.2* and *pck1* play crucial roles in the conversion of non-carbohydrate substrates into glucose [45,46]. Similar results have been confirmed in juvenile GIFT tilapia, where low temperatures elevate the expression levels of G6Pase, g6pc, and PEPCK genes, activating hepatic gluconeogenesis [20,47]. In the liver of the *P. leopardus*, the mRNA levels of *g6pc1a.2* and *pck1* were also significantly upregulated at 13 °C (Figure 5c), suggesting their crucial roles in hepatic gluconeogenesis to respond to the temperature fluctuation.

### 4.3. Hepatic Lipid Peroxidation Damage and Activation of Antioxidant Defense Under Cold Exposure

Exposure to low temperatures not only alters energy metabolism but also triggers lipid peroxidation, which has significant impacts on cellular function [48,49]. At 13 °C, ferroptosis pathways, a type of iron-dependent regulated cell death primarily driven by excessive lipid peroxidation and subsequent membrane damage, were significantly enriched [50]. Under oxidative stress conditions, free radicals produced within the cell attack double bonds in polyunsaturated fatty acids (PUFAs), forming lipid peroxides that disrupt the structure and function of cell membranes, leading to membrane damage [51]. *Acsl4* is a crucial component in the execution of ferroptosis, not only determining the sensitivity to ferroptosis but also serving as a major contributor [52]. The upregulation of *acsl4a* indicates that more unsaturated fatty acids (PUFAs) are incorporated into phospholipids, further triggering lipid peroxidation [53]. Members of the LOX family catalyze the peroxidation of PUFAs, which is particularly important for ferroptosis [54]. The upregulation of *alox15bl* expression signifies the accumulation of lipid peroxidation products and enhances ferroptosis [55]. The upregulation of *tfr1b* suggests an increase in iron uptake, promoting the occurrence of ferroptosis [56]. Damaged cells need to be cleared through apoptosis. In our transcriptomic analysis results, the relative expression levels of positive regulators *acsl4a*, *tfr1*, and *alox15bl* were significantly upregulated, indicating that exposure to low temperatures leads to a more pronounced ferroptosis in the liver.

Once lipid peroxidation occurs in cell membranes, the structural and functional integrity of the membranes is compromised, leading to increased permeability and loss of membrane integrity [57]. This results in the leakage of hepatic enzymes from the cells into the bloodstream [58,59]. GOT and GPT enzyme levels are two critical indicators of liver damage. Our enzyme activity assays showed that while the levels of GOT and GPT enzymes decreased in the liver tissue, their levels increased in the blood, confirming that hepatocytes were damaged and enzyme leakage occurred under low-temperature conditions. Additionally, FISH results also demonstrated significant upregulation of GOT and GPT gene expression in some hepatocytes under low-temperature conditions. GPX enzymes (glutathione peroxidases) are important antioxidants that participate in the clearance of hydrogen peroxide and lipid peroxides [60,61,62]. According to the transcriptomic analysis, GPX genes *gpx1a* and *gpx7* were significantly upregulated. The upregulated expression of these antioxidant enzyme-related genes indicated that the hepatocytes of *P. leopardus* are activating antioxidant defense mechanisms while experiencing oxidative damage.

### 4.4. Upregulation of Genes Related Repair of Liver Damage upon Rewarming

Upon rewarming, it was observed that a significant upregulation of genes involved in the endoplasmic reticulum (ER) protein processing pathway and the proteasomal pathway. The upregulation of numerous genes in the ER protein processing pathway indicated that the cells are undergoing endoplasmic reticulum stress (ERS), a condition induced by disturbances in normal ER function [63]. It has been confirmed that endoplasmic reticulum (ER) stress and proteasomes play a crucial role in the response to cold exposure in teleosts, such as gilthead sea bream, zebrafish, and tiger barb [16,64,65]. Genes involved in multiple processes such as protein folding, modification, transport, and degradation were all upregulated in the liver of the R-25 °C group. Specifically, the upregulation of *atf6* and *edem1* genes serves as a marker for the initiation of the ER stress response [66]. Increased expression of genes such as *hspa5*, *calr*, *uggt1*, *lman2l*, *sec61a1*, and *sec31a* enhances protein folding, modification, and transport [17,67,68]. The upregulation of *derl1*, *syvn1*, and *ube2j1* enhances the recognition and degradation of misfolded proteins [69,70]. Misfolded proteins also led to the upregulation of many genes in the proteasome pathway [71]. As an important mechanism for protein degradation within the cell, the upregulation of numerous genes in the proteasome pathway indicated that the cells are actively addressing the buildup of misfolded proteins or other forms of cellular damage, thereby maintaining protein homeostasis.

## 5. Conclusions

This study explored the impacts of acute cold exposure and subsequent temperature recovery on the liver of *P. leopardus*. Histological analysis and enzyme activity assays demonstrated that temperature fluctuations significantly disrupted normal liver function, leading to lipid accumulation and changes in antioxidant levels. Transcriptomic profiling of liver tissue revealed 2744 differentially expressed genes (DEGs) across three experimental groups: 25 °C (control), 13 °C (cold exposure), and rewarming at 25 °C (R-25 °C). Functional enrichment analysis of these DEGs highlighted significant associations with biological processes, including lipid metabolism and antioxidant defense, as well as key metabolic pathways such as fatty acid biosynthesis and ferroptosis. Cold exposure led to the upregulation of genes related to lipid synthesis and gluconeogenesis in the liver to address metabolic challenges. Cold exposure also induced lipid peroxidation, causing cellular damage, and activated antioxidant defense mechanisms. During rewarming, fatty acid oxidation increased, and pathways involved in endoplasmic reticulum (ER) protein processing and proteasomal activity were upregulated. Additionally, representative DEGs involved in antioxidant activity and lipid metabolism, such as *got1*, *gpx1a*, *gpt*, and *g6pcla.2*, were validated through qRT-PCR and fluorescence in situ hybridization (FISH). Collectively, these findings provide new insights into the physiological adaptations of fish under temperature fluctuations and offer a theoretical foundation for fish farming management and health monitoring.

## Data Availability

The Sequence Read Archive (SRA) has been deposited at GenBank in NCBI, and it will be released upon this study publication. The transcriptome data have been available in the NCBI GenBank repository with the accession number PRJNA1190851; please see the link below for details (https://dataview.ncbi.nlm.nih.gov/object/PRJNA1190851, accessed on 26 November 2024).

## Supplementary Materials

The following supporting information can be downloaded at: https://www.mdpi.com/article/10.3390/antiox14020223/s1, Figure S1: KEGG pathway annotation of differentially expressed genes on *P. Leopardus* in the liver; Figure S2: GO terms enrichment results of DEGs from five clusters in the liver of *P. Leopardus* under temperature changes; Figure S3: Bubble chart of KEGG pathway enrichment analysis of 131 redox-related genes; Table S1: Primer sequences of qPCR; Table S2: Primer sequences of FISH; Table S3: The number of upregulated or downregulated DEGs in each comparison group.
