# Supplementary material for: The Effects of Acute Temperature Changes on Transcriptomic Responses in the Liver of Leopard Coral Groupers (Plectropomus leopardus)
Source: Antioxidants (Basel). 2025 Feb 15;14(2):223. doi: 10.3390/antiox14020223 (PMC11851849; doi:10.3390/antiox14020223)
Supplement: Supplementary file 1 [file antioxidants-14-00223-s001.zip › Table S1.pdf]

Table S1. Primer sequences of qPCR.

	Gene	Primer	Sequences (5′–3′)
qPCR	*got1*	F	AGCTCTTTGCTGAATGGAAGGA
		R	GCTCTGTGATGTGGTCCCAA
	gpt	F	AGGGAACAGTATGGGCTCCT
		R	CATGGTCACAATGCCGTCAC
	gpxla	F	ATCAGTTTGGACATCAGGAGAAC
		R	GCATCATCACTGGGGAATGGA
	cat	F	GGTACTGAACCGGAACCCAG
		R	AGGAGAAAAGACGACCCTGC
	sod2	F	CCTGACCTACGACTATGGCG
		R	TGCCGTCACATCTCCCTTTG
	aox1	F	TCGAGGTCCGTCTCAGTACA
		R	CGGGCTCTCCGATACCCTTT
	g6pcla.2	F	CCCTCTTCTACCTGCTGTCCT
		R	TTCACTTTTCACACGCCCTT
	b2m	F	GTACGGCAAGGACAACACCC
		R	CCTTAACTTTGGTCCCGTGAGT

Note: b2m as the reference gene.
