# Supplementary material for: The Effects of Acute Temperature Changes on Transcriptomic Responses in the Liver of Leopard Coral Groupers (Plectropomus leopardus)
Source: Antioxidants (Basel). 2025 Feb 15;14(2):223. doi: 10.3390/antiox14020223 (PMC11851849; doi:10.3390/antiox14020223)
Supplement: Supplementary file 1 [file antioxidants-14-00223-s001.zip › Table S2.pdf]

Table S2. Primer sequences of FISH.

	Gene	Primer	Sequences (5′–3′)
FISH	*gpxla*	F	ATTTAGGTGACACTATAGGGAACGGCTTTGAACCCAAC
		R	TAATACGACTCATATAGGGTTTACAGCCACCCAGTGCAT
	g6pcla.2	F	ATTTAGGTGACACTATAGCTGCACCTGTTTGACTCCTT
		R	TAATACGACTCACTATAGGGCAGCTCATGTGTTCGTGGTA
	Got1	F	ATTTAGGTGACACTATAGTCGGCTTATCAGGGATTCGC
		R	TAATACGACTCACTATAGGGGCTCTGTGATGTGGTCCCAA
	gpt	F	ATTTAGGTGACACTATAGAAGATCCAAAGGTCTGAGCG
		R	TAATACGACTCACTATAGGGCGTGGGGTTCATAGTGTCGAT

Note: The underlined sequences indicate the binding sites of SP6 and T7 RNA polymerase.
