# Supplementary material for: The Effects of Acute Temperature Changes on Transcriptomic Responses in the Liver of Leopard Coral Groupers (Plectropomus leopardus)
Source: Antioxidants (Basel). 2025 Feb 15;14(2):223. doi: 10.3390/antiox14020223 (PMC11851849; doi:10.3390/antiox14020223)
Supplement: Supplementary file 1 [file antioxidants-14-00223-s001.zip › Table S3.pdf]

Table S3. The number of up-regulated or down-regulated DEGs in each comparison group

Group	Up-regulated	Down-regulated	All	Unique
13°C_vs_25°C	592	767	1359	
R-25°C_vs_13°C	595	725	1320	2744
R-25°C_vs_25°C	477	700	1177	
