# Supplementary figures and images for: The Effects of Acute Temperature Changes on Transcriptomic Responses in the Liver of Leopard Coral Groupers (Plectropomus leopardus)
Source: Antioxidants (Basel). 2025 Feb 15;14(2):223. doi: 10.3390/antiox14020223 (PMC11851849; doi:10.3390/antiox14020223)

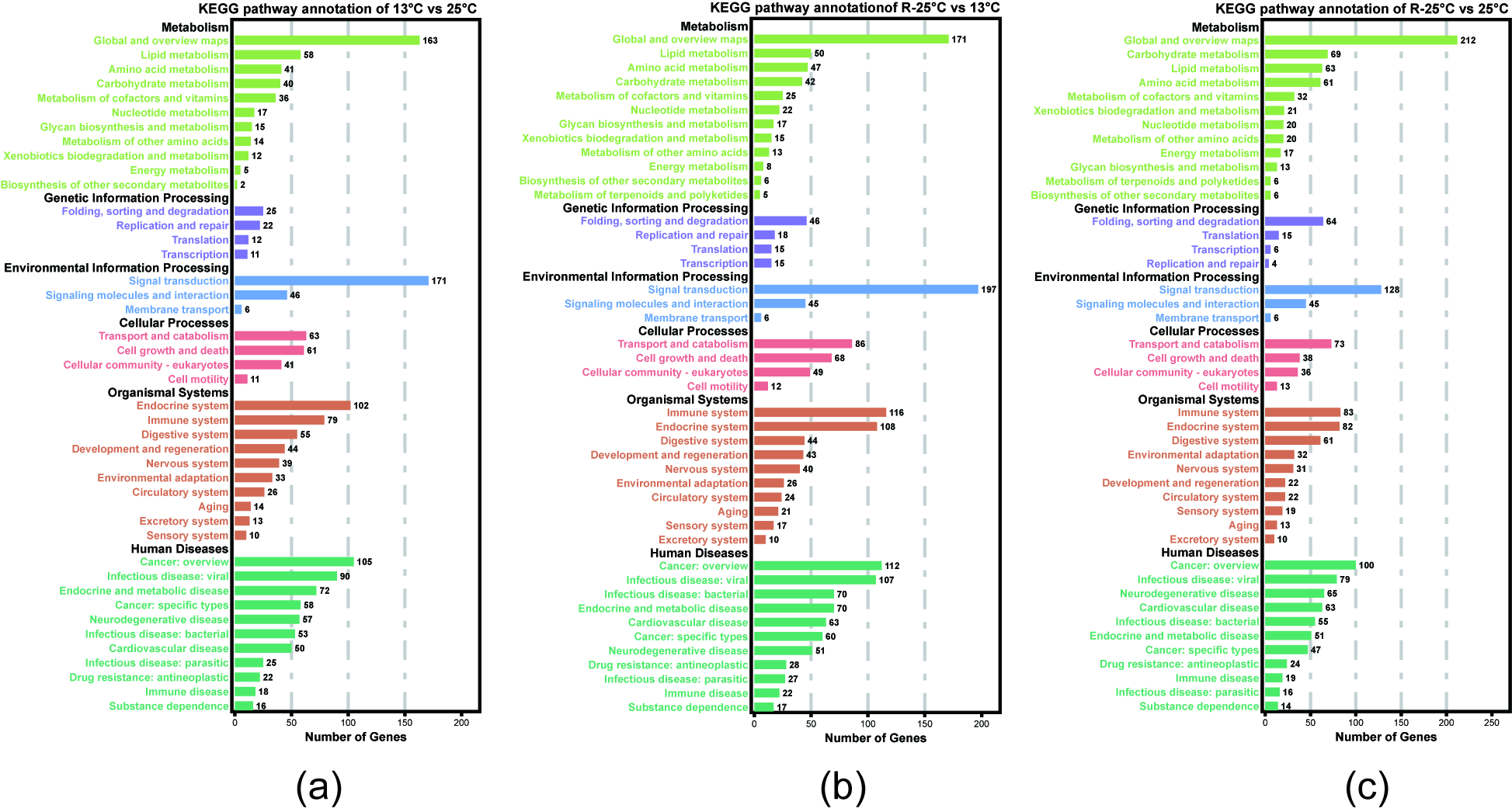

Supplement: Supplementary file 1 [file antioxidants-14-00223-s001.zip › Figure S1.tif]

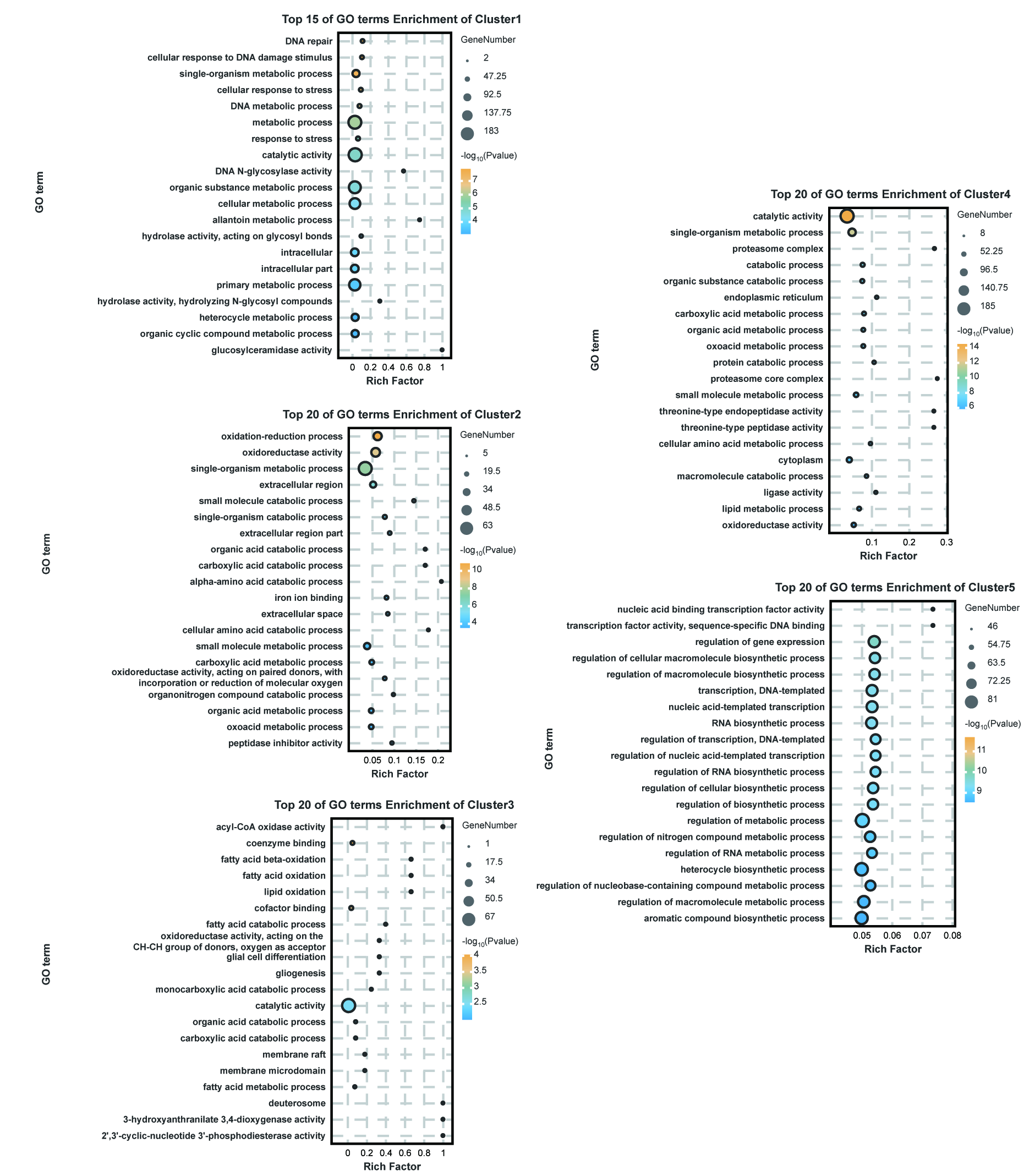

Supplement: Supplementary file 1 [file antioxidants-14-00223-s001.zip › Figure S2.tif]

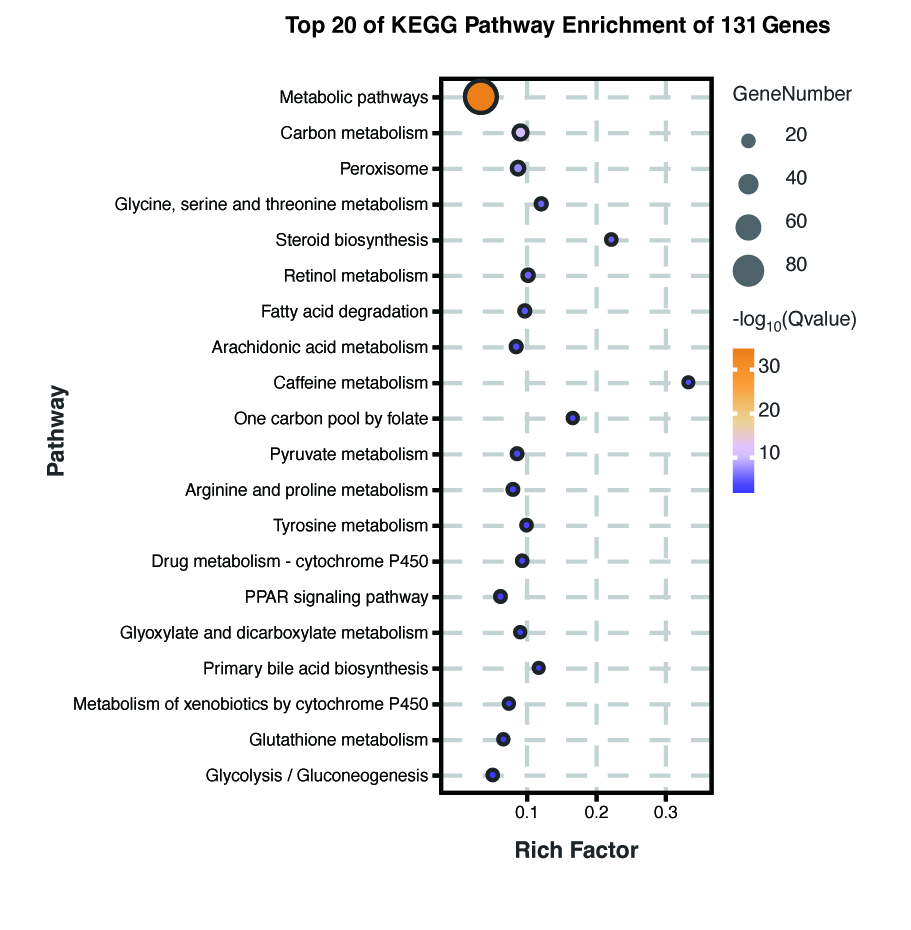

Supplement: Supplementary file 1 [file antioxidants-14-00223-s001.zip › Figure S3.tif]
